# Optimization of Process Variables in the Drilling of LM6/B_4_C Composites through Grey Relational Analysis

**DOI:** 10.3390/ma15144860

**Published:** 2022-07-12

**Authors:** C. Sarala Rubi, J. Udaya Prakash, Robert Čep, Muniyandy Elangovan

**Affiliations:** 1 Department of Physics, Vel Tech Rangarajan Dr. Sagunthala R&D Institute of Science and Technology, Avadi 600062, India; csaralarubi@veltech.edu.in; 2 Department of Mechanical Engineering, Vel Tech Rangarajan Dr. Sagunthala R&D Institute of Science and Technology, Avadi 600062, India; udayaprakashj@veltech.edu.in; 3 Department of Machining, Assembly and Engineering Metrology, Faculty of Mechanical Engineering, VSB-Technical University of Ostrava, 708 00 Ostrava, Czech Republic

**Keywords:** composites, parameters, optimization, drilling, ANOVA

## Abstract

The objective of this investigational analysis was to study the influence of process variables on the response during the drilling of $\mathrm{LM}6/B_{4}C$ composite materials. Stir casting was employed to produce the $\mathrm{LM}6/B_{4}C$ composites. A Vertical Machining Center (VMC) with a dynamometer was used to drill the holes and to record the thrust force. An L27 orthogonal array was used to carry out the experimental work. A grey relational analysis (GRA) was employed to perform optimization in order to attain the lowest Thrust Force (TF), Surface Roughness (SR) and Burr Height (BH). For minimal responses, the optimum levels of the process variables viz. the feed rate (F), spindle speed (S), drill material (D) and reinforcing percentage (R) were determined. The process variables in the drilling of the $\mathrm{LM}6/B_{4}C$ composites were indeed optimized, according to confirmational investigations. The predicted Grey Relational Grade was 0.846, whereas the experimental GRG was 0.865, with a 2.2% error—indicating that the optimization process was valid.

## 1. Introduction

Composites are typically comprised of a polymer matrix, metal matrix or ceramic matrix, based on the physical and chemical properties of the matrix. Metal matrix composites (MMCs) provide numerous benefits over metallic alloys, which include their greater functional strength, high resistance to wear and low coefficient of thermal expansion [1,2,3,4]. The production of composites has therefore been a field of major concern over the last thirty years. The goal in the manufacture of composites is to attain a variety of characteristics that can never be attained in any one of the components alone. The production of composites thus provides an opportunity to modify the characteristics of individual chosen constituents to suit particular requirements. Aluminium Matrix Composites (AMCs) are a type of aluminium-oriented material system that is light and high-performing [5]. Due to their unique properties, composites are commonly used in industry [6]. MMCs have recently received lot more attention than typical metallic materials, and they are now widely used in a variety of commercial applications because of their good strength, light weight, high resistance to wear and exceptional mechanical characteristics [7,8,9,10,11].

The industrial sector demands ultra-lightweight products for load-bearing applications. Therefore, it is essential to substitute with components that would not lead to heavy weights, but which are highly compatible with current economic circumstances, in order to meet current industrial demands. Throughout the field of composites, specifically in AMCs, all these requirements are properly followed. Due to their less-costly existence, AMCs have a wide series of applications in the vehicles, buildings, factories and sports sectors. The most crucial component that affects their characteristics is the physical characteristics of the metal or composite. Microstructural analysis is important when determining performance under prescribed conditions [12]. 

Aluminium—a highly prominent continuous phase used in MMCs—offers a number of benefits, including its light weight, recrystallization strengthening, good corrosion resistance, large thermal and electrical conductivity, machinability and ease of acquisition. Due to these features, it is used in a variety of complicated industry sectors. The lightweight aluminium matrix is coupled with the hardest possible second phase material to obtain AMCs [13,14,15]. MMCs are a kind of composite made up of a continuous metal phase and a conductive or non-metallic second phase material. High hardness, resistance to wear, rigidity and specific strength are the characteristics of these composites; they are preferred over traditional metals owing to their superior characteristics [16]. Particulate-reinforced AMCs are used mostly in the aircraft, military and transportation sectors [17].

MMCs are often produced as a net shape product; however, machining is essential at the finishing stage [18]. Dispersoids play a crucial role in composites and are shaped as particles, fibres or flakes. The second phase material’s primary function is to absorb force in order to strengthen the matrix. The constant integration between the second phase material and the continuous phase creates persistent residual tension in the composite [19].

MMCs can be made in a variety of ways such as powder metallurgy, ball milling, stir casting, pressure casting, etc. The stir casting method for the development of MMCs is commonly adopted because it is inexpensive, huge-scale production is feasible and the second phase material can be dispersed evenly along the matrix [20].

Drilling is the most common machining process for assembly operations. The need for these operations has come into focus for composite materials, which are becoming increasingly common in current times. Composite machining is often a challenging process and impacts the material’s efficiency. $B_{4}C$ is currently used as second phase material for MMC development due to its intrinsic mechanical and chemical characteristics [21,22].

Drilling is employed in aircraft components to provide a good surface finish [23]. Dry drilling is cost effective as it minimizes the costs of material removal, which typically reduces the cost of the product [24]. Furthermore, dry machining requires lower electrical energy than wet machining [25,26]. Additionally, dry machining is environmentally sustainable and has no health risks [27].

## 2. Materials and Methods

The materials used in this study were chosen based on their properties, cost and use. Three sets of composites were fabricated in the form of plates with dimensions of 100 mm× 100 mm × 10 mm and a reinforcement weight percentage of 3, 6 and 9, respectively. The aluminium alloy LM6 was also cast for comparison studies with the composites. The composition of the LM6 alloy is depicted in Table 1 [28].

**Figure 1:**
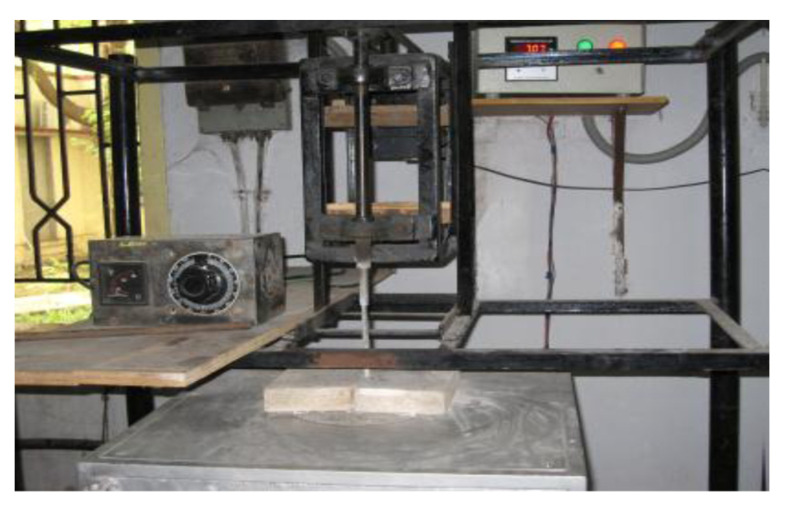
Fabrication of the LM6 alloy using a stir casting setup.

Stir casting is a fluid state process of material manufacturing, where reinforcement is combined with a melted matrix (LM6 alloy) by virtue of mechanical stirring [29]. Figure 1 displays the stir casting setup used for the fabrication process.

### 2.1. Fabrication of the LM6 Alloy/Boron Carbide ($B_{4}C$) Composites

LM6 alloy ingots were heated to 850 °C and melted in an electric furnace. The molten aluminium was mixed well to generate a vortex, and the second phase material ($B_{4}C$) was preheated to (250 °C) and then added. The oxide film that was formed on the surface of the molten metal was removed by adding Titanium (Ti) in the form of Potassium Hexa Fluro Titanate (K_2_TiF_6_; this improves the wettability between the Aluminium alloy and Boron Carbide). The slurry was mixed well at 600 rpm for 10 min for a homogeneous distribution of the ceramic particles. The composite plates, with dimensions of 100 mm× 100 mm× 10 mm, were obtained by transferring the molten mixture to 650 °C pre-heated cast iron moulds and allowing it to solidify.

### 2.2. Drilling

**Figure 2:**
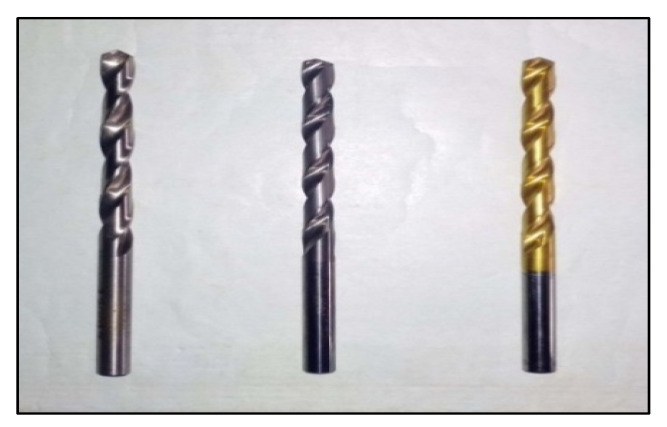
Photograph of drill bits.

**Figure 3:**
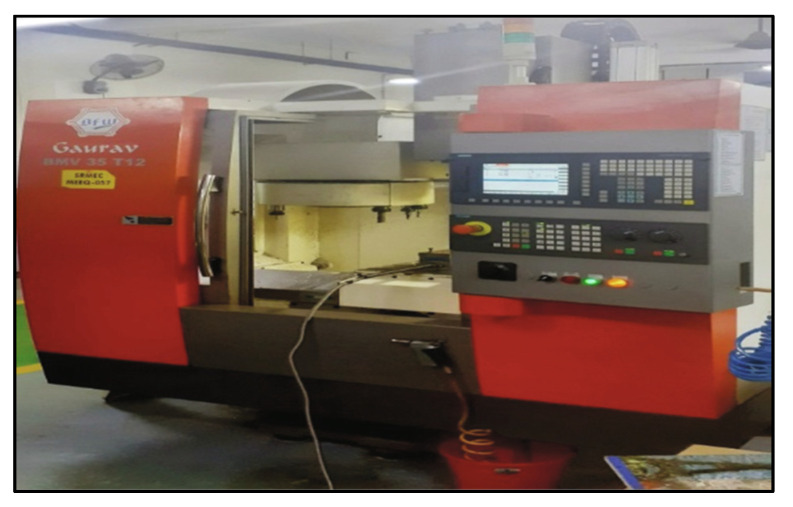
Experimental setup with a dynamometer arrangement.

Drilling MMCs is a useful machining method. The studies here were carried out on a VMC with predetermined process parameters. Data from the experiments was captured and recorded using a computer-based data collecting device [30]. The TF was measured with a Kistler dynamometer. HSS, Carbide and TiN-coated carbide were the three different drill materials used. For all three drills, the diameter, point angle and helix angle was 6 mm, 118° and 30°, respectively. Images of the VMC and drills utilized are presented in Figure 2 and Figure 3. The parameters were chosen based on relevant research as well as trial studies. Table 2 displays the drilling variables and their levels.

### 2.3. Grey Relational Analysis

Multi-criteria decision-making (MCDM) tools are extremely popular for obtaining compromise solutions [31,32,33,34,35,36,37,38]. GRA is much simpler than most MCDMs but performs a similar task, obtaining balanced solutions when multiple conflicting responses are present. GRA was carried out to optimize the process variables in order to obtain the minimum TF, SR and BH. The various steps involved in GRA are described below: (1) The experimental data were initially standardized on a scale of 0 to 1 (2) The grey relational coefficient (GRC) was found with respect to the normal investigational data (3) The GRG was found by averaging the GRC with respect to the TF, SR and BH. The process variable with the highest GRG (Rank 1) was the optimum.

## 3. Measurements

**Figure 4:**
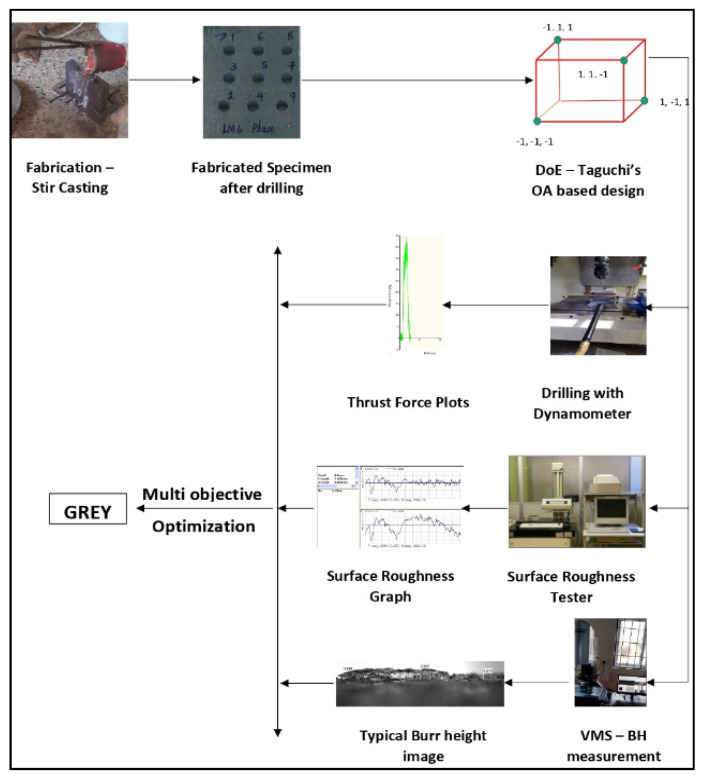
Layout of the experimental setup and measurements.

Figure 4 depicts the layout of the experimental setup and the measurements carried out in this study. 

### 3.1. Thrust Force (TF) Measurement

The response TF was measured in real time utilising a computer-controlled data collecting system and a KISTLER dynamometer, type 9257 B. The TF created while drilling was correspondingly transformed into voltage signals and the data was collected in the form of a graph using the KISTLER Dynoware software.

**Figure 5:**
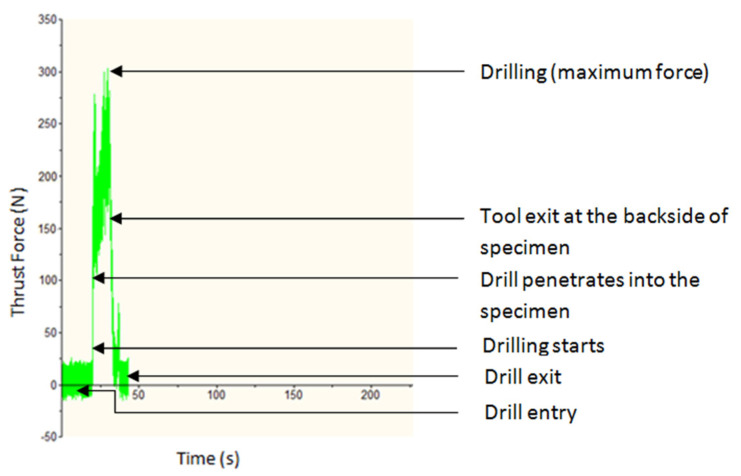
Typical TF measured during the drilling of AMCs.

Figure 5 depicts the usual TF encountered when drilling hybrid MMCs, with the hole-making process divided into six stages. The first stage is the drill’s entry into the MMC, where the TF is minimal, and the second stage is the initiation of the drilling process. The third stage is the penetration of the drill into the specimen, where the hole-making process is completed. The fourth stage denotes the hole-making phase, in which the highest TF is displayed. The fifth stage is the tool’s exit through the back side of the specimen, indicating a significant drop in force. The sixth stage is the drill’s exit from the specimen, where there is no TF. The TF signal acquired in this study was nearly identical to that obtained in previous studies; the variance detected was only in the centre of the hole [39].

### 3.2. Surface Roughness (SR) Measurement

**Figure 6:**
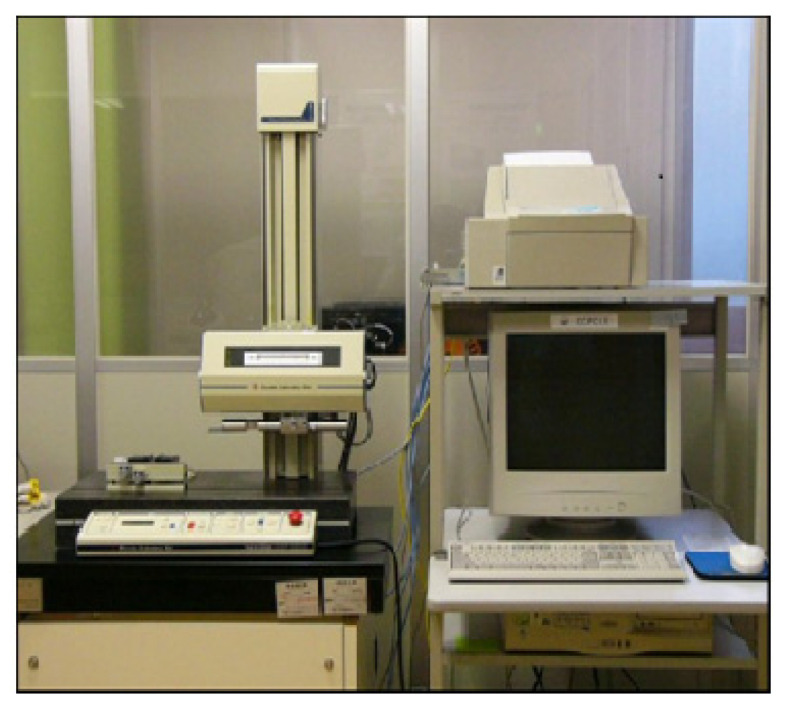
Experimental setup for measuring surface roughness.

The average SR was measured using an SR tester (Surfcorder SE 3500). The data was gathered thrice at various positions on the drilled surface, with the average of the three readings used to calculate the SR. Figure 6 shows the SR tester that was utilised in this study. The direction of a calculation of SR was normal to the drilled hole.

### 3.3. Measurement of Burr Height

**Figure 7:**
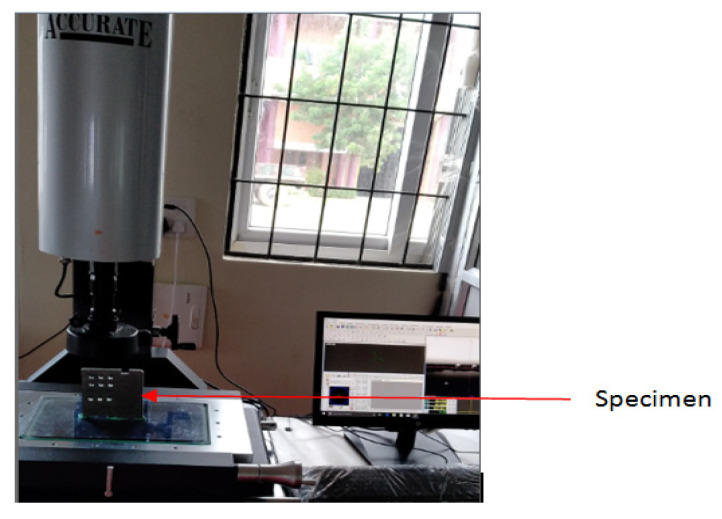
Experimental setup for measuring Burr height.

**Figure 8:**
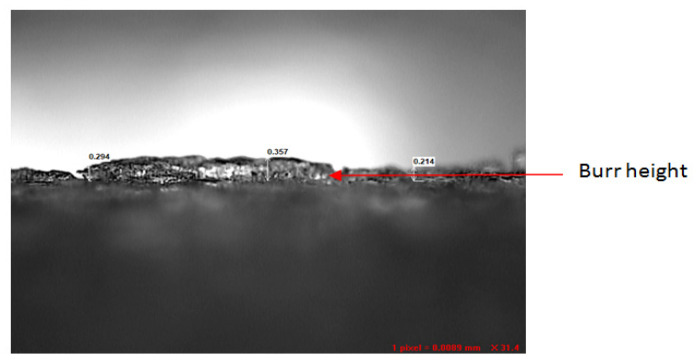
Typical Burr Height observed using a VMS.

A burr is a plastically deformed material created at a drilled hole’s inlet and outlet. These burrs lead to many problems with product performance and quality because they can interconnect during component assembly and can end up causing a jamming effect. At the end of a cut, the development of burrs is equivalent to that of chips. The exit burr is essential, as it is bigger in size and hard to remove and is likely to cause deburring issues. Figure 7 shows the experimental setup for measuring BH. A typical BH observed using a video measurement system (VMS) is shown in Figure 8. The drill exit side of the specimen was kept under the VMS. The exit burr did not have the same height at all points, so the BH was measured at five different places and the average value was used for the analysis. 

## 4. Results and Discussion

### 4.1. Microstructure

**Figure 9:**
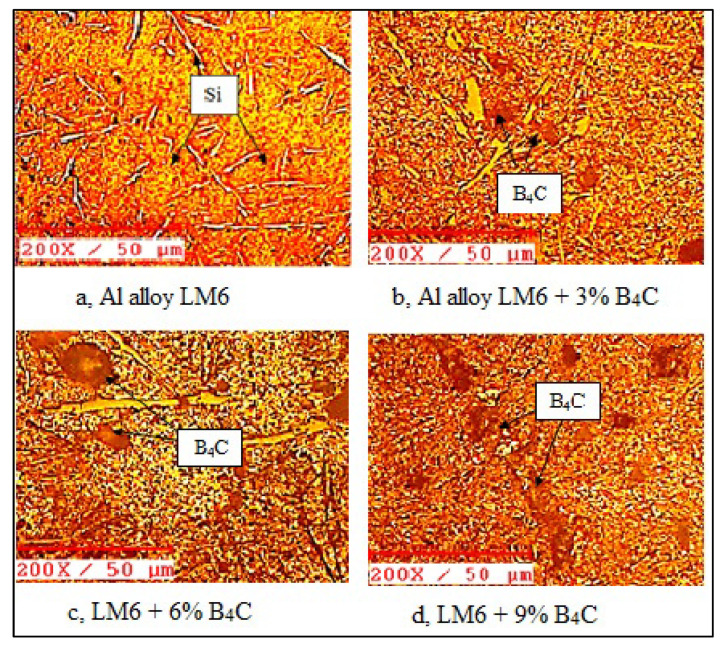
(**a**–**d**) Micrographs of the LM6 alloy and LM6/B_4_C composites.

The main focus of the microstructural analysis was to establish the homogeneous dispersion of the second phase material in the matrix. The composite material Al/$B_{4}C$ was examined using light microscopy. The material was taken out from each composite and precise polishing work was carried out to achieve a reflective surface finish on the material. The optical images of the given composite demonstrate the uniform mixture of the matrix second phase material; the microstructures are displayed in Figure 9.

Figure 9a reveals the microstructures of the LM6 aluminium alloy. The Al–Si eutectic particles look like spikes and scripts, owing to the higher percentage of silicon (Al–Si particles) in the LM6 alloys. Figure 9b reveals the microstructure of the AMCs, with the dispersion of Boron carbide in the LM6 alloy with 3% $B_{4}C$. The composite particles were in close proximity due to the higher fluidity of the LM6, with a higher silicon content and a lower melting point. Figure 9c,d exhibit the microstructures of the MMCs with 6% and 9% $B_{4}C$; an increase in the content of reinforcement particles and the uniform distribution can be seen. 

### 4.2. Hardness

**Figure 10:**
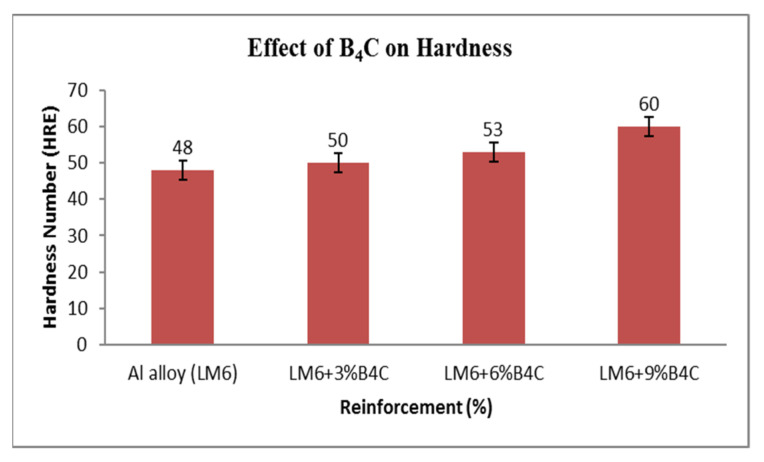
Influence of B_4_C on the hardness of AMCs.

The hardness of the Al $\mathrm{LM}6/B_{4}C$ composites and the LM6 alloy was tested by the Rockwell hardness test scale E (1/8-inch steel ball with a 10 kgf minor load and 90 kgf major load) [1]. It can be seen from Figure 10 that the composite hardness was considerably more than that of the LM6 alloy. Furthermore, the composite’s hardness is known to increase with rises in the wt. percent of boron carbide [40]. The surface areas of the composites were enhanced by implementing the second phase material ($B_{4}C$) into the composite material as well as by reducing the grain sizes of the continuous phase (Al); this prevents plastic deformation. The grain boundaries rose to the highest point, and the misalignment of the atoms was decreased by maximisation of the weight percentage of the second phase material, which increased the matrix strength and thereby increased the composite’s hardness. Kalaiselvan et al. have observed the same phenomenon [41].

### 4.3. Density Measurement

**Figure 11:**
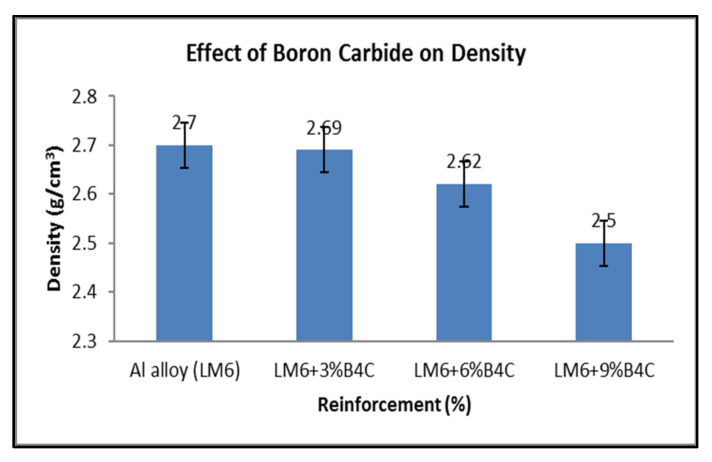
Effect of boron carbide on the density of AMCs.

Displacement techniques are used to measure the density of a material [42]. A 0.001 g-precision electronic weighing balance was used to determine the sample masses. The displaced liquid was used to calculate the volume of the samples. The effect of $B_{4}C$ on the density is shown in Figure 11.

The composite density dropped owing to a rise in the mass percentage of the boron carbide [43]. The reason for this is that boron carbide densities (2.52 g/cm^3^) are smaller than LM6 alloy densities. Monolithic $B_{4}C$ ceramic is a very hard, solid and stiff material with a low density.

### 4.4. Grey Relational Analysis ($LM6/B_{4}C$)

The GRA is used to optimize the process parameters for $\mathrm{LM}6/B_{4}C$ composites, taking into account the different performance factors of the drilling process. To analyse the impact of drilling variables when drilling using VMC, performance features such as F, S, D and R were chosen. Experiments were carried out using the suitable L_27_ OA. 

For the twenty-seven experiments, the GRG was calculated and presented in Table 3. Practically, the greater the grade of the gray relationship, the closer the product would be to the optimum value. Therefore, for optimum performance, a greater GRG is needed. The process variables’ optimum levels are those with the maximum GRG.

**Figure 12:**
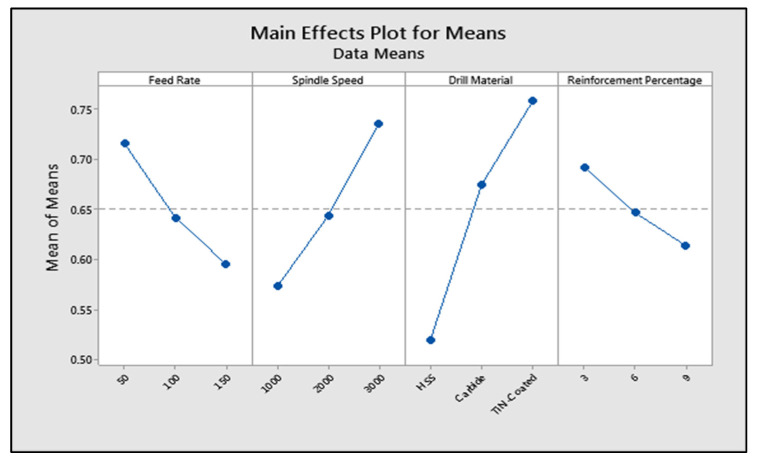
Response Graphs for the GRG (LM6/B_4_C).

The experiments were ranked according to their GRG values. Figure 12 clearly shows that the GRG value was high for the 1st level of ‘F’, the 3rd level of ‘S’, the 3rd level of ‘D’ and the 1st level of ‘R’.

An ANOVA was used to establish the suitable outcome of the process variables in this study by examining their relative contributions to the response [44,45]. The experimental plan carried out was analysed at a 95% level of confidence. Table 4 summarizes the ANOVA of the GRG results. The obtained $R^{2}$ value for the GRG was 93.1%. The *p*-value was smaller than 0.05 for ‘F’ and ‘R’, which indicates that they had a significant impact on the GRG.

Besides the *p* value, it is also possible to use an F-test to decide which process variables have a major impact on the response. As per the Fischer’s F-test, if the obtained F- ratio value is more than the tabulated F-value, it is regarded as significant [46,47,48,49]. It is evident from Table 4 that the F-test values for ‘F’, ‘S’, ‘D’ and ‘R’ were larger than the F-Table values, and hence they had a significant impact on the GRG. Among the chosen process variables, the drill material (51.44%) had the maximum contribution on the GRG, followed by the spindle speed (23.29%), feed rate (12.92%) and reinforcement percentage (5.45%). The contributions of the interactions were much less, and so they were pooled with the error term.

The ANOVA table shows the relative contribution of each process variable on the GRG values. The degrees of freedom (DoF) are obtained by subtracting one from the number of levels. The total DoF is the number of experiments minus one. SS means the sum of squares. The Mean Square (MS) is given by its sum of squares divided by its degrees of freedom; this is also known as the variance, which is why the test is known as the analysis of variance (ANOVA). 

### 4.5. Confirmation Experiments

To determine the optimum parameters, the investigational outcomes were analysed. From Figure 12, the optimum process variables for attaining the maximum GRG (minimum TF, SR and BH) were the variables at levels F_1_, S_3_, D_3_, R_1_, which were an ‘F’ of 50 mm/min, an ‘S’ of 3000 rpm, a TiN-coated drill bit and a 3% $B_{4}C$ particulate. The predicted GRG was 0.846, whereas the experimental GRG was 0.865. A good agreement attained with respect to the predicted and experimental values can be seen, and the error was 2.2%, so the methodology of optimization held well.

### 4.6. The Effects of Drilling Process Variables on the GRG

**Figure 13:**
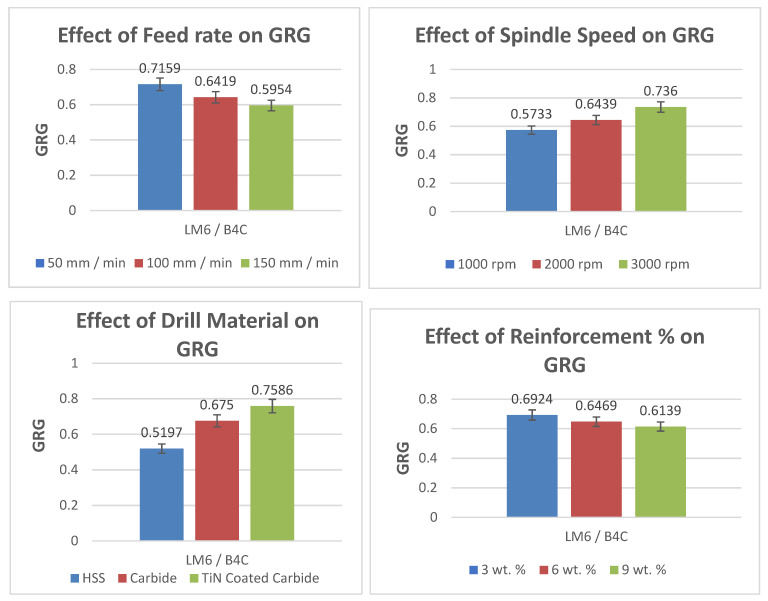
Effect of drilling process parameters on the GRG.

The effects of the process parameters on the GRG (Figure 13) are based on response Table 5. The GRG values of all 27 experiments were calculated and the level 1 value in Table 5 is the average of nine level 1 values; level 2 is the average of nine level 2 values and level 3 is the average of nine level 3 values. 

For example, the feed rate value of 50 m/min was the average of the first nine values (Ex No. 1–9); in the same way, the feed rate value of 100 m/min was the average of the second nine values (Ex No. 10–18) and the feed rate value of 150 m/min was the average of the third nine values (Ex No. 19–27). For example, the spindle speed value of 1000 rpm was the average of Ex Nos. 1, 2, 3, 10, 11, 12, 19, 20 and 21.

The GRG was employed as a quality representation of all the responses, including the TF, SR and BH. The process effect of the variables on the GRG is shown in Figure 13. The highest value of the GRG response graph suggests that drilling variables had a stronger impact on machinability features [50,51]. The peak value of the GRG was obtained at an ‘F’ of 50 mm/min, an ‘S’ of 3000 rpm, a ‘D’ of TiN-coated and an ‘R’ % of 3 wt. %; this are the optimal process parameters for drilling.

It was observed that the highest GRG was obtained at the lowest ‘F’ and highest ‘S’, which implies that the response TF, SR and BH were minimal at the lowest ‘F’ and highest ‘S’. This is because friction between the drill bit and the specimen is decreased when the ‘F’ is decreased, resulting in a lower TF. A lower ‘F’ reduces the temperature generated during drilling, which improves the surface quality. It was observed that a lower ‘F’ gave a lower TF, which gave a good surface finish at a smaller feed. 

Due to the rapid heat rise generated by friction at greater spindle speeds, the work piece softens and penetrates smoothly, leading to a smaller TF. As spindle speed increases, the cutting time is reduced, which results in a reduced thrust force and reduced work piece distortion and, hence, the surface finish is improved; this provides better GRG values. 

The SR value for 3% $B_{4}C$ was less than that of the other two wt %. In the $\mathrm{LM}6/B_{4}C$ composite material, the TF value was larger with the addition of $B_{4}C$. The physics behind this phenomenon is that $B_{4}C$ is the hardest material—so by increasing the $B_{4}C$%, the composite’s hardness rises as well, which results in a larger TF. The SR of the $\mathrm{LM}6/B_{4}C$ decreases initially when the weight percentage of the reinforcement increases.

As the ‘F’ rises, so does the cutting force and BH. For high feed rates, the BH is the largest. The BH decreases with rises in the reinforcement % for $\mathrm{LM}6/B_{4}C$ composites. The GRG value is based on the average of the GRC of the TF, the GRC of the SR and the GRC of the BH of each experiment; so, the GRG value depends on the response TF, SR and BH.

## 5. Conclusions

Drilling experiments on $\mathrm{LM}6/B_{4}C$ composites were conducted to analyse the impact of drilling process variables such as the feed rate, spindle speed and drilling material, as well as the reinforcement percentage. The experimental and statistical analyses led to the following results:

- $\mathrm{LM}6/B_{4}C$ composites were prepared by the low cost Stir casting method.
- The uniform distribution of the second phase material in the matrix was confirmed by Optical micrographs.
- The densities of the $\mathrm{LM}6/B_{4}C$ composites decreased with rises in the wt. % of the $B_{4}C$, whereas the hardness increased with increases in the reinforcement.
- Drilling experiments were conducted on $\mathrm{LM}6/B_{4}C$ composites using Taguchi’s DoE and analysed using Grey relational analyses.
- The TF, SR and BH values decreased with decreases in the feed rate for all the specimens.
- The TF, SR and Burr height values decreased with rises in the spindle speed for all the specimens.
- The TiN-Coated carbide drill bit provided the optimum Surface Roughness and Burr Height values for all the composites.
- The predicted GRG was 0.846, whereas the experimental GRG was 0.865. A good agreement attained with respect to the predicted and experimental values could be seen and the error was 2.2%, so the methodology of optimization held well.

## Figures and Tables

**Table 1:** Elemental composition of the LM6 alloy.

Constituent	Si	Cu	Fe	Mg	Mn	Ti	Ni	Zn	Al
Wt. %	11.48	0.013	0.52	0.02	0.01	0.02	0.01	0.01	Remainder

**Table 2:** Drilling variables and levels.

Level	F (mm/min)	S (rpm)	D	R %
1	50	1000	HSS	3
2	100	2000	Carbide	6
3	150	3000	TiN-Coated	9

**Table 3:** Grey Relational Analysis (LM6/B_4_C).

Expt. No.	F (mm/min)	S (rpm)	D (Drill Material)	R (wt. %)	GRC of TF	GRC of SR	GRC of BH	GRG	Rank
1	50	1000	HSS	3	0.769	0.355	0.564	0.563	18
2	50	1000	Carbide	6	0.76	0.518	0.775	0.684	11
3	50	1000	TiN-Coated	9	0.602	0.739	0.898	0.746	9
4	50	2000	HSS	6	0.649	0.383	0.623	0.552	21
5	50	2000	Carbide	9	0.587	0.543	0.975	0.702	10
6	50	2000	TiN-Coated	3	1	0.825	1	0.942	1
7	50	3000	HSS	9	0.766	0.447	0.627	0.613	14
8	50	3000	Carbide	3	0.772	0.992	0.703	0.822	5
9	50	3000	TiN-Coated	6	0.708	1	0.75	0.819	6
10	100	1000	HSS	3	0.582	0.349	0.491	0.474	26
11	100	1000	Carbide	6	0.599	0.505	0.564	0.556	20
12	100	1000	TiN-Coated	9	0.493	0.715	0.75	0.653	13
13	100	2000	HSS	6	0.561	0.373	0.603	0.512	24
14	100	2000	Carbide	9	0.565	0.528	0.683	0.592	15
15	100	2000	TiN-Coated	3	0.73	0.813	0.726	0.756	8
16	100	3000	HSS	9	0.643	0.438	0.613	0.565	17
17	100	3000	Carbide	3	0.615	0.958	0.935	0.836	2
18	100	3000	TiN-Coated	6	0.6	0.949	0.951	0.833	4
19	150	1000	HSS	3	0.478	0.333	0.333	0.381	27
20	150	1000	Carbide	6	0.557	0.471	0.594	0.541	22
21	150	1000	TiN-Coated	9	0.333	0.649	0.703	0.562	19
22	150	2000	HSS	6	0.511	0.368	0.594	0.491	25
23	150	2000	Carbide	9	0.56	0.494	0.644	0.566	16
24	150	2000	TiN-Coated	3	0.594	0.71	0.741	0.682	12
25	150	3000	HSS	9	0.572	0.413	0.594	0.526	23
26	150	3000	Carbide	3	0.592	0.838	0.898	0.776	7
27	150	3000	TiN-Coated	6	0.672	0.896	0.935	0.834	3

**Table 4:** ANOVA for GRG (LM6/B_4_C).

Source of Variation	DoF	SS	MS	F	*p*	Contribution (%)
Feed Rate (F)	2	0.066	0.033	16.84	0.00	12.92
Spindle Speed (S)	2	0.120	0.060	30.37	0.00	23.29
Drill Material (D)	2	0.265	0.132	67.07	0.00	51.44
Reinforcement Percentage (R)	2	0.028	0.014	7.1	0.01	5.45
Pooled Error	18	0.036	0.002			6.90
Total	26	0.514				100.00

**Table 5:** Response Table for GRG (LM6/B_4_C).

Level	F	S	D	R
1	0.7159	0.5733	0.5197	0.6924
2	0.6419	0.6439	0.675	0.6469
3	0.5954	0.736	0.7586	0.6139
Delta	0.1204	0.1627	0.2389	0.0786
Rank	3	2	1	4

## Data Availability

The data presented in this study are available through email upon request to the corresponding author.
